# Understanding the Use of Smartphone Apps for Health Information Among Pregnant Chinese Women: Mixed Methods Study

**DOI:** 10.2196/12631

**Published:** 2019-06-18

**Authors:** Na Wang, Zequn Deng, Li Ming Wen, Yan Ding, Gengsheng He

**Affiliations:** 1 Department of Nutrition and Food Hygiene School of Public Health Fudan University Shanghai China; 2 Nursing Department Obstetrics and Gynaecology Hospital of Fudan University Fudan University Shanghai China; 3 Key Laboratory of Public Health Safety of Ministry of Education Fudan University Shanghai China; 4 School of Public Health University of Sydney Sydney Australia; 5 Health Promotion Unit Sydney Local Health District Sydney Australia

**Keywords:** mobile applications, pregnancy, consumer health information, health promotion

## Abstract

**Background:**

Hospital-based health promotion resources to assist pregnant women in adopting a healthy lifestyle and optimizing gestational weight gain are important, but with limited effects. Increasingly, women are using mobile apps to access health information during the antenatal period.

**Objective:**

The aims of the study were to investigate app-usage by Chinese women during pregnancy and to gain a better understanding of their views and attitudes toward apps containing health information.

**Methods:**

A mixed methods study design was applied. Study participants were recruited from 2 maternity hospitals in Shanghai, China, between March and July 2018. A self-administered Web-based survey was conducted with 535 pregnant Chinese women on their sources of health information and reasons for using apps during pregnancy. A total of 4 semistructured focus groups were also conducted with the pregnant women (n=28).

**Results:**

The use of pregnancy-related apps and the internet was common among the respondents. Almost half of the women had used pregnancy-related apps. Specifically, the use of apps for health information declined as pregnancy progressed from 70% (35/50) in the first trimester to 41.3% (143/346) in the third trimester. The main reason for using an app was to monitor fetal development (436/535, 81.5%), followed by learning about nutrition and recording diet in pregnancy (140/535, 26.2%). The women found that the apps were useful and convenient and can support lifestyle modifications during pregnancy. However, some apps also contained misinformation or incorrect information that could cause anxiety as reported by the participants. Many women expressed the need for developing an app containing evidence-based, well-informed, and tailored health information to support them during pregnancy.

**Conclusions:**

The study suggests that apps were widely used by many Chinese women during pregnancy to monitor fetal development, to obtain diet and physical activity information, and to track their body changes. The women highly appreciated the evidence-based information, expert opinions, and tailored advice available on apps. Smartphone apps have the potential to deliver health information for pregnant women.

## Introduction

### Background

The Developmental Origins of Health and Disease concept describes how, during early life (at conception and/or during fetal life, infancy, and early childhood), the environment induces changes in development that have a long-term impact on later health and disease risk [1]. Maternal lifestyle, such as diet and physical activity, and their health status (eg, obesity and gestational diabetes mellitus) can affect the potential risk of metabolic diseases, obesity, and diabetes of the next generation [2,3]. In general, women are especially receptive to advice about a healthy lifestyle during pregnancy, in which optimizing their lifestyle for the health benefits of their offspring is a powerful motivator. Despite this, physical inactivity, under nutrition or over nutrition, and suboptimal gestational weight gain (GWG) are commonly reported by studies with pregnant women [4-6].

Pregnancy is a life phase when women are adjusting to their new status as mothers. They may be dealing with uncertainty and anxiety, as well as learning how to care for themselves and their newborns [7]. Hospital-based resources to assist pregnant women in achieving better nutrition and physical activity levels and optimizing GWG are extremely important, but with limited effects [8,9]. Research also suggests that many people would seek health information via Web-based media following a medical visit when they felt dissatisfied or disagreed with the advice they received from their medical providers [10]. However, seeking more health information can also be positive; people may look for additional and complementary information to discuss with health care professionals.

Mobile health (mHealth), defined as the use of mobile phones and other wireless technology such as texting messaging, apps, and video messaging to support the achievement of health objectives [11], is a burgeoning field of public health. With the widespread application of digital health care, mHealth services are also thriving in the field of maternal and child health in China [12]. More women are turning to digital sources for health information and support during pregnancy and early motherhood [13]. In particular, thousands of maternal and child health-related apps have appeared in the Android app market and app store in China to cater to women’s needs [12]. These apps cover a range of topics relating to maternal and fetal health, from general factual encyclopedias of pregnancy facts to more specific information on maternal diet for gestational diabetes [14]. Recently, a survey conducted in Hubei province, China, showed that women used apps mainly to monitor and record fetal development and their body changes as well [15]. However, gaps exist between functions of apps in the current market and women’s expectations for functions of a pregnancy app. These gaps mainly reflect in the ability of an app to solve the common problems encountered and its guidance on diet and physical activity during pregnancy. Hearn et al conducted a study with perinatal women in Australia, which was focused on developing a website and an app for health promotion during the perinatal period [16]. Their study found that, in particular, women wanted information on issues, such as nutrition and diet, exercise, managing weight gain, sleeping problems, emotional fluctuations, allergies, breastfeeding, and gestational diabetes.

Smartphone apps may provide a novel way to provide health information, facilitate individual access to resources, and increase health care engagement. They could be used as a strategy to overcome some barriers associated with traditional face-to-face health care interactions [14]. Some commonly cited barriers to traditional health care include lack of referrals from health care professionals, program availability, inconveniences of meetings (eg, lack of time or transportation), patient embarrassment, and high financial costs [17]. Recently, several studies have investigated the use of apps as a tool to prevent excessive GWG and to manage or prevent gestational diabetes and some of them demonstrated the potential feasibility and efficacy of app-based interventions in promoting a healthy lifestyle in the antenatal period [18-20]. A systematic review [20] found that most studies on mHealth apps that aim to support lifestyle and medical care for high-income countries revealed the usability of these apps to reduce GWG, to increase intake of vegetables and fruit, to quit smoking but still needed further evidence to show the effective use of these apps during pregnancy. Ledford’s study showed that, as a prenatal education tool, mobile apps effectively improved pregnant women’s self-management for health [19].However, despite the increasing use of apps by pregnant women, to date, few published studies have focused on how and why women used apps for health promotion during pregnancy, particularly in the Chinese context.

### Objectives

The aims of this study were to (1) investigate app usage of Chinese women during pregnancy, (2) gain a better understanding of their views and attitudes toward apps, (3) explore their concerns about data privacy and security, and (4) inform the development of a health promotion program through an app.

## Methods

### Study Design

A mixed methods study was used with both quantitative and qualitative studies, consisting of a cross-sectional Web-based survey and focus group (FG) interviews. In the survey, we focused on women’s current status of app usage during pregnancy and their intention to use an app for health promotion information. To obtain a better understanding of pregnant women’s attitudes and views of using apps, semistructured FGs were conducted. Both the survey and FGs were conducted in Shanghai, China, between March and July 2018.

### Setting and Participants

This study was conducted in 2 maternity hospitals, the Obstetrics and Gynecology Hospital of Fudan University and Maternal and Child Health Hospital, Changning District, Shanghai, China. The former is a university-affiliated maternity hospital, a tertiary hospital with the total number of births around 15,000 annually. The latter is a district hospital ranked as a secondary hospital with the total number of births being 11,000 annually. Hospitals in China are categorized as primary, secondary, or tertiary institutions according to a 3-tier system that recognizes a hospital’s capacity to provide medical care and medical education and conduct medical research. For example, a tertiary hospital is responsible for providing specialist health services and performing a bigger role with regard to medical education and scientific research.

The purpose of recruiting pregnant women from the tertiary and secondary hospitals was to have a broader representative study sample. All the pregnant women who had registered in the clinics were required to attend the routine antenatal clinics and 5 face-to-face sessions of antenatal education throughout their pregnancy. At the end of the antenatal education sessions, women were approached by 2 research nurses for their eligibility and consent.

Women were eligible if they were (1) aged 18 years and above, (2) currently pregnant, (3) owning a smartphone, and (4) able to give written consent to participate.

### Ethical Considerations

The study was approved by the Ethics Committee of the Obstetrics and Gynecology Hospital of Fudan University (Ethics Approval number 2017-74). Before conducting the survey or FGs, all potential participants were informed about the purposes and procedures of the study, and their written informed consent was obtained from those agreeing to participate. All participants were notified that they had the right to refuse to participate, and in addition, they could subsequently withdraw from the study at any time if they had participated.

### Data Collection

#### A Cross-Sectional Survey

A self-administered Web-based survey using iPads was undertaken by pregnant women during their clinical visits using the survey website . A total of 582 women were approached by 2 research nurses for their eligibility and consent for the survey, and 535 respondents provided informed consent and completed the survey with a completion rate of 91.9% (535/582). The main reason for refusal was *not interested* or *uncompleted questionnaires*. To check for completeness, the mandatory items of the questionnaire would be highlighted. Participants were able to review and change their answers through a Back button before submission. No incentives were provided for participation in this survey.

The 19-item questionnaire (Multimedia Appendix 1) included maternal sociodemographic characteristics, medical history and current pregnancy information, health behaviors, and smartphone and app usage during pregnancy. The questionnaire was developed in consultation with experts in the field and pilot tested in 20 respondents before the survey. Physical activity levels during pregnancy were assessed based on the International Physical Activity Questionnaire [21]. In our study, we followed the American College of Obstetricians and Gynecologists (ACOG) guidelines [22] for physical activity for pregnant women to evaluate their daily moderate activity levels. The last 4 questions were about smartphone and pregnancy app usage. These questions had multiple response options, and the respondents could also add other options for the questions. These survey items were sourced from other surveys [15,23] for pregnancy app usage and were adapted for our survey.

#### Focus Groups

Following the Web-based survey, the women were invited to participate in the FG interviews if they indicated their interest in having further discussion on their views of using an app in the Web-based survey. Women who consented to the interview were purposively recruited to obtain a sample with a wide range of gestational ages. A total of 50 pregnant women were approached to participate in the FGs. Of them, 28 (28/50, 56%) agreed to participate, with their ages ranging between 24 and 37 years and gestational ages ranging between 13 and 40 weeks. During the FG session, the first author, as the moderator, conducted all the interviews with assistance from the second author (ZD) in a conference room located at the Obstetrics and Gynecology Hospital of Fudan University. The FGs were conducted in Mandarin and tape-recorded. A semistructured interview guide was used during the group discussion to ensure that all required information was sought [24], but the discussion remained open to any new issues or concerns related to app usage for supporting pregnancy. The interview guide with open-ended questions was developed based on the Web-based survey and a literature review of similar studies. The FGs began with broad issues and were then narrowed to more specific topics as the group discussion progressed. The main discussion questions covered topics including the following:

1. Their experience, attitudes, and views regarding using an app for supporting pregnancy;
2. Their sources of health information and reasons for selecting a particular app for pregnancy;
3. Their concerns about data privacy and security issues when using the apps;
4. What features they would like to see and use in an app for supporting pregnancy.

Each FG consisted of 5 to 8 women and took about 40 to 60 min. During the group discussion, field notes were also taken to facilitate the data analysis. Data saturation was reached after 4 FGs when no further unique or new themes were introduced by the FG participants.

### Data Analysis

For the cross-sectional survey, data were analyzed using the SPSS software package (version 22.0, SPSS Inc). Body mass index (BMI) was calculated as weight (kg)/height (meters) squared and classified as underweight (BMI <18.5 kg/m^2^), normal weight (BMI 18.5 to 23.9 kg/m^2^), overweight (BMI 24.0 to 27.9 kg/m^2^), or obese (BMI ≥28.0 kg/m^2^) based on the BMI cut points for the Chinese [25]. Women were grouped into adequate GWG, inadequate GWG, and excessive GWG according to the Institute of Medicine (IOM) recommendations for week-specific weight gain during pregnancy [26]. Descriptive statistics were used to present the characteristics of the respondents, providing frequencies (numbers) and proportion (percentages) for categorical variables. Continuous variables were described with mean and SD or median and interquartile range (IQR) in the case of abnormal distribution. Women’s sources of information and reasons for using apps during pregnancy were analyzed and compared between different trimesters using the chi-square test.

A multivariable logistic regression was used to calculate adjusted odds ratios (ORs) and 95% CIs to identify factors associated with app usage of pregnant women in Shanghai. A few steps were taken to identify these factors. First, we used forward stepwise regression to remove those variables with *P* values >.20. Second, considering that education and house income were related to app usage as shown by previous studies [7,23], we also included these 2 variables in the models. In addition, maternal age as a potential confounder was also included in the final model. Finally, we included maternal age, education, household income, parity, prepregnancy BMI category, and trimester as independent variables in the multiple regression model.

Qualitative data from the 4 FGs were used to supplement the quantitative findings. All audio recordings were transcribed verbatim following each FG within 24 hours. Transcripts and field notes were reviewed and analyzed by 2 independent reviewers using a thematic analysis [27]. The goal of a thematic analysis is to identify themes, that is patterns in the data, that are important or interesting and use these themes to address the research or say something about an issue. We performed the analysis based on Braun and Clarke’s 6-phase framework for doing a thematic analysis (Multimedia Appendix 2) [28]. After reading and rereading our transcript, we became familiar with our data. We worked through each transcript and coded every segment of the text that seemed to be relevant to or specifically address our research question. Specifically, line-by-line coding highlighted key comments depicting the attitudes and views regarding using apps and its related factors. Codes were then organized into broader themes that seemed to express something specific about our research question. Finally, we gathered together all the data that were relevant to each theme to review and refine the emerging themes. Any discrepancies in data analysis between the 2 authors involved were resolved by discussion until a consensus had been reached on the codes, subthemes, and themes.

## Results

### Quantitative Findings

#### Characteristics of The Survey Respondents

The demographic characteristics of the study population are presented in Table 1. The mean maternal age was 30.6 (SD 3.6 years) and the median (IQR) gestational age was 32 (17 to 33) weeks. Of the 535 respondents, 478 (478/535, 89.3%) were nulliparous. The prevalence of overweight and obesity was 19.4% (104/535), whereas underweight women were accounted for 15.9% (85/535). Compared with the IOM recommendations for GWG, only 43.7% of women had normal GWG. Only 22.1% (118/535) of women had a physical activity level meeting the guideline recommended by ACOG for physical activity for pregnant women. Anemia was also common among our population (23.7%, 127/535). The percentage of women smoking before pregnancy was 3.7% (20/535), but all quit during pregnancy. The respondents as a whole were well educated, with more than 90% (500/535, 93.5%) having completed a college degree.

**Table 1:** Characteristics of study participants in the survey, Shanghai, China, 2018 (N=535).

Characteristics		Values
**Age (years), n (%)**		
	≤25	30 (5.6)
	26-34	436 (81.5)
	≥35	69 (12.9)
**Education, n (%)**		
	≤Senior high school	35 (6.5)
	College degree	398 (74.4)
	Postgraduate degree or above	102 (19.1)
**Marital status, n (%)**		
	Single/divorced/separated/widowed	6 (1.1)
	Married	529 (98.9)
**Household income^a,b^, n (%)**		
	<¥10,000	115 (21.8)
	¥10,000-¥30,000	302 (57.2)
	>¥30,000	111 (21.0)
**Health issues and physical activity, n (%)**		
	First degree family history of diabetes mellitus	73 (13.6)
	Anemia before or during pregnancy	127 (23.7)
	Smoking before pregnancy	20 (3.7)
	Smoking during pregnancy	0 (0)
	Physical activity (moderate level <30 min)	417 (77.9)
	Parity (primipara)	478 (89.3)
**Prepregnancy weight category, n (%)**		
	Underweight	85 (15.9)
	Normal weight	346 (64.7)
	Overweight	75 (14.0)
	Obese	29 (5.4)
Gestational week (week), median (interquartile range)		32 (17-33)
**Weight gain, n (%)**		
	Inadequate GWG^c^	172 (32.1)
	Adequate GWG	234 (43.7)
	Excessive GWG	129 (24.1)

^a^N=528.

^b^One Chinese Yuan (¥)=US $0.1437.

^c^GWG: gestational weight gain.

#### App Usage and Sources of Health Information During Pregnancy

Table 2 shows the respondents’ sources of health information during pregnancy. More than 80% (438/535, 81.9%) of the respondents’ smartphone operating system was iOS. Women reported that they received pregnancy-related health information mostly from Web-based media (73.3%, 392/535), followed by pregnancy apps (49.2%, 263/535), face-to-face consultations with health professionals (20.4%, 109/535), family and friends (18.1%, 97/535), and print materials (15.5%, 83/535), whereas their preferred or expected sources of information were Web-based media (68.4%, 366/535), followed by pregnancy apps (48.8%, 261/535) and face-to-face consultations with health professionals (34.4%, 184/535). We further compared the difference between women’s expected sources of information and their current information sources. The results showed that the actual source of information from health professionals was lower than that expected by the pregnant women (*P*<.001), whereas the source of information from family/friends was higher than that which was expected (*P*=.001). In addition, women’s reported sources of information varied over the period of their pregnancy. Specifically, the use of an app for health information declined as pregnancy progressed from 70% (35/50) in the first trimester to 41.3% (143/346) in the third trimester. A logistic regression analysis also showed that, compared with those in the third trimester, the rates of app usage for health information were significantly higher in the first trimester (adjusted OR 3.057, 95% CI 1.575-5.932) and the second trimester (adjusted OR 2.206, 95% CI 1.453-3.349). In addition, nulliparous women were more likely to use an app for health information (adjusted OR 1.975, 95% CI 1.043-3.742; Table 3).

**Table 2:** Sources of health information by trimester of pregnancy of Chinese women in Shanghai, China, 2018.

Sources of health information		All women (N=535), n (%)	First trimester (n=50), n (%)	Second trimester (n=139), n (%)	Third trimester (n=346), n (%)	*P* value
**Smartphone operating system**						
	iOS	438 (81.9)	39 (78)	111 (79.9)	288 (83.2)	.52
	Android	97 (18.1)	11 (22)	28 (20.1)	58 (16.8)	.52
**Expected sources of information for health promotion**						
	Pregnancy apps	261 (48.8)	35 (70)	75 (54.0)	151 (43.6)	.001^a^
	Other Web-based media	366 (68.4)	37 (74)	96 (69.1)	233 (67.3)	.63
	Television	36 (6.7)	6 (12)	17 (12.2)	13 (3.8)	.001^a^
	Paper materials	84 (15.7)	14 (28)	33 (23.7)	37 (10.7)	<.001^a^
	Face-to-face with health professionals	184 (34.4)	23(46）	57（41.0）	104（30.1）	.01^a^
	Family/friends	59 (11.0)	4 (8)	4 (2.9)	51 (14.7)	.001^a^
**Current sources of information for health promotion**						
	Pregnancy apps	263 (49.2)	35 (70)	85 (61.2)	143 (41.3)	<.001^a^
	Other Web-based media	392 (73.3)	40 (80)	120 (86.3)	232 (67.1)	<.001^a^
	Television	39 (7.3)	8 (16)	17 (12.2)	14 (4.0)	<.001^a^
	Paper materials	83 (15.5)	15 (30)	35 (25.2)	33 (9.5)	<.001^a^
	Face-to-face consultations with health professionals^b^	109 (20.4)	6 (12)	27 (19.4)	76 (22.0)	.25
	Family/friends^b^	97 (18.1)	3 (6)	5 (3.6)	89 (25.7)	<.001^a^

^a^Represents a significant difference between the 3 groups.

^b^There were significant differences between current sources of information and expected sources of information from face-to-face consultations with health professionals (*P*<.001) and family/friends (*P*=.001) for health promotion.

**Table 3:** Results of a multivariable logistic regression analysis of factors associated with app usage of pregnant women in Shanghai, China (N=528; after exclusion of 7 cases for missing data on household income).

Factors^a^		Beta	SE	Wald *X*^2^	Odds ratio	95% CI	*P* value
**Age (years)**		—^b^	—	0.286	—	—	.87
	≤25	—	—	—	1.000 (reference)	—	—
	26-34	.041	0.400	0.010	1.042	0.475-2.282	.92
	≥35	−.113	0.474	0.057	0.893	0.352-2.263	.81
Education (1= Postgraduate degree or above, 0=≤College degree)		.036	0.238	0.024	1.037	0.651-1.653	.88
**Household income^c^**		—	—	3.787	—	—	.15
	<¥10,000	—	—	—	1.000 (reference)	—	—
	¥10,000-30,000	.222	0.233	0.910	1.249	0.791-1.970	.34
	>¥30,000	.559	0.290	3.722	1.749	0.991-3.087	.05
Parity (1=multipara, 0=primipara)		.681	0.326	4.361	1.975	1.043-3.742	.04^d^
**Prepregnancy weight category**		—	—	6.994	—	—	.07
	Underweight	.197	0.256	0.594	1.218	0.738-2.010	.44
	Normal weight	—	—	—	1.000 (reference)	—	—
	Overweight	−.396	0.264	2.248	0.673	0.401-1.129	.13
	Obese	−.952	0.486	3.843	0.386	0.149-1.000	.05
**Trimester**		—	—	20.736	—	—	<.001^d^
	First	1.117	0.338	10.91	3.057	1.575-5.932	.001^d^
	Second	.791	0.213	13.802	2.206	1.453-3.349	<.001^d^
	Third	—	—	—	1.000 (reference)	—	—

^a^Regression models included maternal age, education, household income, parity, pre-pregnancy body mass index category and trimester.

^b^Not applicable.

^c^One Chinese Yuan (¥)=US $0.1437.

^d^Represents the variable is significant in the logistic regression model.

As shown in Table 4, the most reported reason of app usage was for monitoring fetal development (436/535, 81.5%). Getting information related to nutrition and recording diet during pregnancy (26.2%, 140/535) was also a frequently reported reason for using an app. Other reasons for app usage included keeping track of information about their antenatal care appointments (128/535, 23.9%), tracking their own body (101/535, 18.9%), getting information related to physical activity and recording exercise during pregnancy (91/535, 17.0%), recording antenatal examination (87/535, 16.3%), having Web-based discussions with other pregnant women (83/535, 15.5%), storing photos of themselves during pregnancy (52/535, 9.7%), and uploading and storing fetal ultrasound images (27/535, 5.0%). These reasons for using apps during pregnancy also changed over the period of pregnancy.

**Table 4:** Reasons for using pregnancy apps by the trimester of pregnancy.

Reasons for using pregnancy apps	All women (N=535), n (%)	First trimester (n=50), n (%)	Second trimester (n=139), n (%)	Third trimester (n=346), n (%)	*P* value
Monitoring fetal development	436 (81.5)	36 (72)	89 (64.0)	311 (89.9)	<.001^a^
Tracking own body	101 (18.9)	16 (32)	37 (26.6)	48 (13.9)	<.001^a^
Learning information regarding nutrition during pregnancy and recording diet	140 (26.2)	16 (32)	48 (34.5)	76 (22.0)	.01^a^
Learning information regarding physical activity during pregnancy and recording exercise	91 (17.0)	14 (28)	25 (18.0)	52 (15.0)	.07
Understanding the content of antenatal care	128 (23.9)	15 (30)	40 (28.8)	73 (21.1)	.12
Storing photos of themselves	52 (9.7)	4 (8)	8 (5.8)	40 (11.6)	.14
Storing fetal ultrasound images	27 (5.0)	3 (6)	6 (4.3)	18 (5.2)	.88
Recording antenatal examination	87 (16.3)	7 (14)	18 (12.9)	62 (17.9)	.37
Web-based discussions with other pregnant women	83 (15.5)	7 (14)	21 (15.1)	55 (15.9)	.93

^a^Represents a significant difference between the 3 groups.

**Table 5:** Demographic characteristics of focus group participants in Shanghai, China.

Characteristics		Group 1 (n=5)	Group 2 (n=8)	Group 3 (n=7)	Group 4 (n=8)	Total (n=28)
Age (years), mean (SD)		29.8 (3.5)	29.3 (3.4)	30.1 (1.7)	29.3 (3.8)	29.6 (3.1)
**Household income^a^, n (%)**						
	<¥10,000	2 (40)	4 (50)	2 (29)	3 (38)	11 (39)
	¥10,000-30,000	1 (20)	4 (50)	4 (57)	5 (63)	14 (50)
	>¥30,000	2 (40)	0 (0)	1 (14)	0 (0)	3 (11)
**Education, n (%)**						
	≤Senior high school	1 (20)	0 (0)	0 (0)	2 (25)	3 (11)
	College degree	3 (60)	6 (75)	5 (71)	6 (75)	20 (71)
	Postgraduate degree or above	1 (20)	2 (25)	2 (29)	0 (0)	5 (18)
Parity (primipara), n (%)		4 (80)	8 (100)	6 (86)	8 (100)	26 (93)
Prepregnancy body mass index (kg/m2), median (IQR^b^)		18.6 (16.9-18.9)	21.8 (20.4-27.4)	22.6 (20.8-22.4)	20.6 (18.5-22.3)	21.0 (18.9-23.5)
Gestational age (week), median (IQR)		32 (13-36)	33 (15-40)	30 (18-37)	34 (17-39)	32 (15-38)

^a^One Chinese Yuan (¥)=US $0.1437.

^b^IQR: interquartile range.

### Qualitative Findings

The characteristics of FG participants are shown in Table 5. In total, 4 overarching descriptive themes emerged from the data analysis (Multimedia Appendix 3) about app usage during pregnancy, including (1) accompany and support, (2) disadvantages, (3) data privacy and security issues, and (4) expectations for features of an app. Participants’ quotations were used to illustrate the original text, and only codes were used to maintain anonymity.

#### Theme 1: Accompany and Support

##### Usefulness

In general, most of the participants (n=26) regarded that apps or other Web-based media play an indispensable role during the period of pregnancy, especially in their early stage of pregnancy. Participants valued apps with multifunctional features:

Most of the features are useful, such as what you can eat and what you can’t eat during pregnancy, interpretation of baby ultrasound report, and how to deal with discomfort during pregnancy, and so on.

Focus Group (FG) 1, Participant (P): C

Some participants (n=3) used apps before pregnancy and continued to use apps after pregnancy. These behaviors reflected that apps were useful and helpful in their daily life. Apps provided them reference, companionship, and support:

I used “Crazy Creation” before pregnancy, it helped me conceive. Now I use “Pregnancy Butler Pro” to accompany pregnancy.

FG2, P: C

Participants (n=13) also valued the discussion forum platform provided by mobile apps, through which they could gain peer support, especially from experienced women or *someone who has just experienced this process*, who help a lot in their discussion forum:

I like online discussion and exchange feelings with other pregnant women and it can offer me some tips for my pregnancy and make me reassurance.

FG3, P: A

##### Convenience

The convenient nature of accessing health information through apps was favorably compared with the waiting time in clinics and the trip to a hospital for talking to health care professionals. Women sought apps and other Web-based media, partly because the antenatal care visit structure and the information delivered during antenatal care visits could not meet their needs. These needs were often reflected in questions such as “Do I need to take nutritional supplements?”, “How do I deal with insomnia and constipation conditions during pregnancy?”, and “Can I take medicines when I get a cold?”. Women also expressed their understanding of doctors’ heavy workload as doctors lacked time to deal with their queries. In China, Maternity Health Care is an obstetrician-led model, where there is no role for midwives to provide a health promotion service, which is different from many other countries. This became evident during the FGs as shown in their statements (n=10):

Usually, I can see doctors once a month for less than ten minutes conversation every time. Just imaging when I suffered from insomnia, the app and internet would provide me very detailed information regarding how to improve sleep immediately...You can see the advantages of it.

FG4, P: B

Another woman echoed this opinion:

Doctors like to say there is no other way than prescribing medicine. In that case, I don’t want to bother him/her...I prefer to search the answers on my app.

FG4, P: D

##### Supporting Women’s Lifestyle Modifications During Pregnancy

Pregnancy is widely viewed as a period when women are open to lifestyle changes. In our study, all the participants said they would change their behaviors that may harm their babies’ health. To reach optimal pregnancy outcomes, an app can facilitate their lifestyle changes for a healthy pregnancy (n=20):

Yes, all the foods and exercise would be checked through app and internet. Sometimes, I can’t help eating McDonald’s, but it was really reduced a lot...I am also using the app to record my daily eating, physical activity and weight gain...the app can provide me a weight-gain curve, it is interesting.

FG2, P: C

#### Theme 2: Disadvantages in Apps

##### Negative Emotions

Although learning about the experiences of others may help individuals feel at ease, some women pointed out that they felt more worried and anxious after having read information on discussion forums through apps . Throughout the FG discussions (n=10), there was acknowledgment that many of the stories people posted on apps were often *horror stories*:

The grievances in some of the forums are too heavy, (discussions on) “Baby Tree” (one of apps) is full of negative emotions...Today a miscarriage of information suddenly jumped in front of me...no one want to see information like this.

FG1, P: C

##### Commercial Motives

As apps in the Chinese market can be freely downloaded by everyone, many developers would take advantage of this opportunity to make profits through advertisements:

There are advertisements in the “Baby Tree”...many contents are charged.

FG1, P: A

Even though women valued expert advice and expressed the desire for information and support offered by health care professionals, they did not want to pay for these Web-based services; however, they accepted the idea of patients paying for medical services in the hospital settings (n=8):

When I want to seek expert advice and some detailed explanation on this topic via apps...I am interested after scanning, but I was pointed in... you should pay for it.

FG1, P: C

##### Invalidated Information

Most pregnant women (n=18) were worried about the lack of a scientific and validated source of information available on apps:

I don’t like anything about the messy apps in the app store, the information in the “Baby Tree” is quite contradictory sometimes, for example, the app that I used before said leeks and red beans could cause miscarriage, but it still recommended the recipe of red bean porridge to me...

FG4, P: G

#### Theme 3: Privacy and Data Security

Pregnant women (n=8) across all FGs said they were not worried about information security issues, because they were willing to sacrifice some privacy or personal information to gain their convenience and other uses. It was mentioned in 2 FGs (n=4) that when the hospital and developer cooperate to develop a pregnancy app, the hospital should be responsible for the privacy and security of the pregnant women’s information:

You shouldn’t find a place for information security right now, unless you don’t use your phone or the internet.

FG3, P: F

#### Theme 4: Expectations for Features of an App

##### Professional and Evidence-Based Information

Participants (n=22) mentioned that an ideal app should provide them staged information and professional advice based on gestational age or different trimesters. When childbirth is approaching, they want to learn something about infant feeding practice in advance:

Apps should provide symptoms during each trimester that a (pregnant) women should have and give gestation-specific information...If only apps can tell me baby care such as breastfeeding and how to handle problems that may encountered.

FG4, P: A and FG2, P: E

I hope that the article can come from the doctor's hand, it is more convincing...Don’t be confrontational (contradictory) between articles.

FG4, P: G

However, some women (n=4) had a different opinion toward information given by doctors. They wanted a gold standard or evidence-based guide that could be referenced and directed, rather than just doctors’ opinions. The information acquired through apps helped them to take control of themselves:

Doctors have different opinions on the same report...one doctor told me to eat iron supplement, but the other said that I didn’t need...it made me confused. I hope that an app provides the reference then I can read my report myself.

FG3, P: B

##### Generality and Individuality

Women wanted information from general factual encyclopedias of pregnancy facts to more specific domains. Particularly, when women suffered from complications during pregnancy, they desired an app to support disease management for conditions, such as gestational diabetes. This means that an app is expected to not only provide general information related to pregnancy but also give personalized support based on the individuals’ conditions (n=6):

Can the app set up a diabetes zone? To tell the truth, I don't know how to eat after diagnosis of gestational diabetes until hospitalization...There are too many diabetes mums.

FG3, P: G

##### Integrating With Antenatal Care

Participants (n=10) favored integrating pregnancy apps with their antenatal care in the hospital and further receiving personalized advice from health professionals:

It is best to have an app that can be connected to the medical card and linked to the usual check-ups, then give suggestions based on personalized examinations and different gestational weeks. This is relatively better than anything else.

FG4, P: E

## Discussion

### Principal Findings

This study provides in-depth insights into the app usage of Chinese women during pregnancy. Our findings suggest that the use of apps and the internet during pregnancy was common among the respondents. Women found apps to be useful and convenient for accessing health information and can be used to promote lifestyle modifications during pregnancy. However, some apps also contained misinformation or incorrect information that could cause anxiety, confusion, and misguidance. The need for developing an app containing evidence-based, well-informed, and tailored health information was also expressed by many women in our study.

Pregnancy is a period that includes many changes which require physical, psychological, and social adaptation. The majority of the pregnant women in our study were first-time mothers, and they needed more information to cope with these changes during pregnancy. They sought information from various sources, such as their health providers, apps and the internet, books, and family and friends. Pregnant women are increasingly using mobile apps as a source of supplemental information [29]. In our study, nearly half of the women stated that they used at least one mobile app during pregnancy. Other studies reported that the rate of use of pregnancy-related mobile apps ranged from 41.3% to 73% [7,12,23,30], and this wide variability may relate to their local contexts, socioeconomic status, the time of the survey, gestational age, and parity. Researchers suggest that websites and apps may be particularly helpful for women from socioeconomically disadvantaged groups, who may lack access to traditional media sources [31].

Our survey also found that women’s usage of apps declined from the first trimester to third trimester, which was also supported by the FGs indicating that they particularly needed pregnancy apps or other sources of information to guide them on what to do in their early pregnancy. Similar findings were also reported by Lupton and Pedersen [23]. In addition, women may turn to other sources for support, such as experienced women within their family or friend circle, as we found that the reported source of information from family and friends increased as pregnancy progressed.

Similar to findings from surveys conducted in Australia and Turkey [23,32], most women participating in this study used pregnancy apps to monitor fetal development, whereas only 26.2% and 17.0% of women used apps for managing their diets and exercise, respectively. A number of participants in the FGs expressed that they wanted to know what they can or cannot eat during pregnancy. The lower rate of using these functions may be due to a lack of evidence-based and credible information available on apps [33]. Womack pointed out that only 50% of their study sample cited pregnancy apps as a source for their recommendation and presented conflicting recommendations for alcohol and food intake during pregnancy. This lack of credibility alongside simultaneous conflicts of risk threatens women’s ability to discern which recommendations should be followed [34]. Kaimal reported in their study of Web-based resources for obstetrics that only 3.6% of websites were created or sponsored by obstetrician-gynecologists [35]. In our FGs, information offered by health professionals was highly appreciated. When encountered with contradictive information between apps or between the app and their health professionals, they tended to believe what their doctors said. They expressed the need for information of future apps to be provided by health professionals. However, doctors may also have different opinions toward the same medical report. As a highly educated group in our study, some women called for the *gold standard*, so they can judge right or wrong depending on the standard information. The information available on apps can empower them to actively engage in monitoring their own health.

In addition, participants in this study favored more interactive apps and recommended communication platforms for both pregnant women and medical staff. Client-to-client communication could meet the demand of users to seek peer support by communicating with women who had similar experiences or health issues. Users can share their experiences and knowledge with others and also gain emotional, social, or practical support from others [36]. However, it is hard to confirm the reliability of the information shared by experienced women. It is also possible that learning from the experiences of others on the Web may lead to greater anxiety or confusion about one’s own condition [37], as reflected by our study. Lee showed that although a social networking function was important for pregnant women, interaction with health professionals remained limited [30]. This may be due to women’s attitudes toward paying fees for consultations with health professionals.

Furthermore, most study participants in this study wanted pregnancy apps to have a Web-based linkage with hospital information systems so that more personalized and tailored information is provided by the apps in a convenient manner, without the need of women having to enter information such as their medical reports. However, most hospital information systems are closed networks; it will be really challenging to achieve free data transfer between apps and existing hospital electronic systems, given the concerns over data security and privacy issues. A recent survey, conducted in China, based on the app market showed that only a small number of apps had an internet connection with hospital information systems to support making appointments, making payments, obtaining hospital service guidance, or checking laboratory results [12], but without providing personalized information or suggestions. It is also noted that, in our FGs, no one expressed concerns about data privacy and security issues when using pregnancy apps. However, they pointed out that it should be the hospitals’ responsibility to ensure data security. We believe that data privacy and security issues are always important in the digital world. Legal frameworks that govern the integrity of health data transfer and storage, in addition to identifying access control and medical liability, are critical to enabling mHealth in China [11].

Finally, it is worth mentioning that a considerable proportion of women in the survey suffered from anemia and abnormal prepregnancy BMI. In addition, more than half of the respondents gained abnormal GWG and over 70% did not reach the goal of physical activity levels recommended by the ACOG guideline [22], indicating poor maternal health status in this study population. This calls for the need for more effective interventions to support maternal and child health in the metropolitan area in China. To the best of our knowledge, this study was the first to investigate women’s lifestyles, GWG, and their use of pregnancy apps simultaneously. Technological platforms, such as smartphone apps, have the unique capacity to integrate different functional modules to manage antenatal health. Recent studies conducted in some Western countries have demonstrated the effectiveness of an app-based lifestyle intervention on GWG and behavior changes [38]. However, in this complex and equivocal information environment, women are often provided with invalidated information by various prenatal apps [34]. This may limit the benefits that women would have got if apps containing quality content and evidence-based information were available.

### Limitations

There were a few limitations in the study. First, the survey was completed by women from 2 hospitals in Shanghai, thus the findings may not be generalizable to pregnant women in other settings. In addition, prepregnancy BMI and GWG classification were based on self-reported body weight and height, in addition to self-reported anemia. We also acknowledge some potential limitations regarding the validity of the questions included in the survey. Furthermore, only women’s views and attitudes were captured regarding their use of apps during pregnancy. Future studies should explore the views of health care professionals or policymakers regarding the feasibility or acceptability of an app for supporting women during pregnancy in the Chinese context.

### Conclusions

The study provides insights into how pregnant women used apps for health information and their views and attitudes toward the apps. It suggests that apps were widely used by many pregnant women to monitor fetal development, to get diet and physical activity information, and to track their body changes. Women highly appreciated evidence-based information, expert opinions, and tailored advice available on the apps. With an increasing number of women using apps during pregnancy, development of quality apps may have the potential to improve prenatal outcomes by facilitating access to health information, reducing demands for face-to-face health services, and enabling the provision of personalized care. Efforts should be made by health professionals, app developers, and policymakers to ensure quality apps be developed for providing timely health promotion information. The apps can be integrated into maternal care to meet the needs of pregnant women, although it is extremely important to ensure privacy and security of individuals’ data.

## Multimedia Appendix 1

Survey questions used in the cross-sectional survey.

## Multimedia Appendix 2

Braun and Clarke’s 6-phase framework for doing a thematic analysis.

## Multimedia Appendix 3

Codes, subthemes, and themes derived from the focus groups.

## Abbreviations

ACOG: American College of Obstetricians and Gynecologists
BMI: body mass index
FG: focus group
GWG: gestational weight gain
IOM: Institute of Medicine
IQR: interquartile range
mHealth: mobile health
OR: odds ratio
