# Supplementary material for: Understanding the Use of Smartphone Apps for Health Information Among Pregnant Chinese Women: Mixed Methods Study
Source: JMIR Mhealth Uhealth. 2019 Jun 18;7(6):e12631. doi: 10.2196/12631 (PMC6604500; doi:10.2196/12631)
Supplement: Supplementary file 1 [file mhealth_v7i6e12631_app1.pdf]

Multimedia Appendix 1 Survey questions used in the cross-sectional survey

Sociodemographics

1. What is your date of birth?
   ______ / _______ / _______
   day month year

2. What is your educational level?
   ☐ ≦Senior high school
   ☐ College degree
   ☐ Master degree or above

3. What's your present marital status?
   ☐ Married
   ☐ Never married
   ☐ Separated but not divorced
   ☐ Divorced
   ☐ Widowed

4. Before tax is taken out, which of the following best describes your approximate household monthly income range from all sources?
   ☐ Less than 10000 yuan;
   ☐ 10000-30000 yuan
   ☐ More than 30000 yuan
   (one Chinese Yuan = US $ 0.1437)

Medical and family history; and pregnancy conditions

5. How tall are you without shoes? __________(cm)

6. About how much did you weigh before you became pregnant? __________(kg)

7. About how much do you weigh now? __________(kg)

8. What is your gestational ages? __________(weeks)

9. How many children have you given birth to?___________

10. Do you have a first degree family history of diabetes mellitus (including your mother, your father and your brothers or sisters)?
    ☐ No ☐ Yes

11. Do you have anemia before or during pregnancy?
    ☐ No ☐ Yes

Health behaviors

12. Had you ever smoked within 3 months before pregnancy:

☐ No ☐ Yes

13. Have you ever smoked during pregnancy?

☐ No ☐ Yes

14. Have you ever drinking alcohol during pregnancy?

☐ No ☐ Yes

15. Physical activity levels during pregnancy:

1) In the last week, how much time did you spend per day walking for recreation, exercise or to get to or from places?

☐ Less than 30min
☐ 30-60min (including 30min)
☐ 60-120min (including 60min)
☐ More than 120min

2) In the last week, how much time did you spend per day doing moderate physical activities (e.g. gentle swimming, social tennis, yoga, brisk walking)?

☐ Less than 30min
☐ 30-60min (including 30min)
☐ 60-120min (including 60min)
☐ More than 120min

3) In the last week, how much time did you spend per day doing vigorous physical activity which made your breathe harder or puff and pant (e,g. jogging, cycling, competitive tennis)?

☐ Less than 30min
☐ 30-60min (including 30min)
☐ 60-120min (including 60min)
☐ More than 120min

4) In the last week, you average daily sitting time

☐ Less than 2 hours
☐ 2-8 hours (including 2h)
☐ 8-12 hours (including 8h)
☐ More than 12 hours

5) In the last week, your average daily sleeping time ____________hours

Smartphone and pregnancy app usage

16. What is your smartphone operating system?

☐ iOS ☐ Android

17. Which of the following are your expected or preferred sources of information for health promotion during pregnancy?
☐ Pregnancy apps
☐ Other online media
☐ Television
☐ Paper materials
☐ Face to face with health professionals
☐ Family/friends
☐ Others__________

18. Which of the following are your current sources of information for health promotion?
☐ Pregnancy apps
☐Other online media
☐ Television
☐ Paper materials
☐ Face to face with health professionals
☐ Family/friends
☐ Others ___________

19. Which of the following are your reasons or purposes for using pregnancy apps?
☐ Monitoring fetal development
☐ Tracking own body
☐ Learning information regarding pregnant nutrition and recording diet
☐ Learning information regarding pregnant physical activity and recording exercise
☐ Understanding the content of antenatal care
☐ Storing photos of woman
☐ Storing fetal ultrasound images
☐ Recording antenatal examination
☐ Online discussions with other pregnant women
☐ Others ___________
