# Supplementary material for: Understanding the Use of Smartphone Apps for Health Information Among Pregnant Chinese Women: Mixed Methods Study
Source: JMIR Mhealth Uhealth. 2019 Jun 18;7(6):e12631. doi: 10.2196/12631 (PMC6604500; doi:10.2196/12631)
Supplement: Supplementary file 2 [file mhealth_v7i6e12631_app2.pdf]

Multimedia Appendix 2 Braun & Clarke's six-phase framework for doing a thematic analysis

Phase		Description of the process
1.	Familiarizing yourself with your data:	Transcribing data (if necessary), reading and re-reading the data, noting down initial ideas.
2.	Generating initial codes:	Coding interesting features of the data in a systematic fashion across the entire data set, collating data relevant to each code.
3.	Searching for themes:	Collating codes into potential themes, gathering all data relevant to each potential theme
4.	Reviewing themes:	Checking if the themes work in relation to the coded extracts and the entire data set, generating a thematic 'map' of the analysis.
5.	Defining and naming themes:	Ongoing analysis to refine the specifics of each theme, and the overall story the analysis tells, generating clear definitions and names for each theme.
6.	Producing the report:	The final opportunity for analysis. Selection of vivid, compelling extract examples, final analysis of selected extracts, relating back of the analysis to the research question and literature, producing a scholarly report of the analysis.
