# Supplementary material for: Understanding the Use of Smartphone Apps for Health Information Among Pregnant Chinese Women: Mixed Methods Study
Source: JMIR Mhealth Uhealth. 2019 Jun 18;7(6):e12631. doi: 10.2196/12631 (PMC6604500; doi:10.2196/12631)
Supplement: Supplementary file 3 [file mhealth_v7i6e12631_app3.pdf]

Multimedia Appendix 3 Codes, subthemes and themes derived from focus groups.

Codes	Subthemes	Themes
Advertisement & Shopping<br>Consultation and peer support<br>Reference for general problems faced during pregnancy<br>Track the development of the baby<br>Learn knowledge in advance<br>Listen to prenatal music<br>Self-monitoring<br>Accompany<br>Detailed information	Usefulness	Accompany and support
Reference for general problems faced during pregnancy<br>Convenience<br>Choose the best suit app<br>Don't need go to hospital<br>Search information with immediate feed back	Convenience	
Lifestyle reference : food, nutrients, and diet; physical activity	Supporting lifestyle modifications	
Grievances<br>Miscarriage	Negative emotions	Disadvantages
Too much advertisement<br>Be charged	Commercial motives	
Not professional<br>Contrast information<br>By experience	Invalidated information	
Don't care about data security<br>You cannot find a place for information security right now, unless you do not use your phone or go online.<br>The hospitals' responsibility for data security		Data privacy and security issues
Professional advice<br>Come from the doctor's hand<br>Gold standard or evidence-based guide	Professional and evidence-based information	Expectations for features of an app

General information<br>Give personalized support based on individuals' conditions	Generality and individuality
Be connected to the medical card<br>Be linked to the usual check-ups<br>Giving suggestions based on personalized examinations.	Integration with antenatal care
